# Erythroferrone, Hepcidin, and Erythropoietin in Chronic Kidney Disease: Associations with Hemoglobin and Renal Function

**DOI:** 10.3390/jcm14217789

**Published:** 2025-11-02

**Authors:** Kürşad Öneç, Gülşah Altun, Şeyma Özdemir Aytekin, Fatih Davran, Birgül Öneç

**Affiliations:** 1 Department of Nephrology, Faculty of Medicine, Duzce University, 81600 Duzce, Turkey; gulsahaltuntas11@gmail.com; 2 Department of Internal Medicine, Faculty of Medicine, Duzce University, 81600 Duzce, Turkey; seyma_ozdemir171@hotmail.com; 3 Department of Biochemistry, Faculty of Medicine, Duzce University, 81600 Duzce, Turkey; fatihdavran@hotmail.com; 4 Department of Hematology, Faculty of Medicine, Duzce University, 81600 Duzce, Turkey; birgulonec@gmail.com

**Keywords:** chronic kidney disease, anemia, erythroferrone, hepcidin, erythropoietin

## Abstract

**Background/Objectives**: Chronic kidney disease (CKD) is commonly complicated by anemia resulting from impaired erythropoietin (EPO) production, iron dysregulation, and chronic inflammation. Erythroferrone (ERFE) and hepcidin are key regulators of erythropoiesis and iron metabolism, but their interaction in CKD remains incompletely understood. This study aimed to examine the associations among ERFE, hepcidin, EPO, and hemoglobin, and to determine whether these markers independently relate to anemia severity in CKD. **Methods**: This cross-sectional case–control study included 126 patients with CKD (stages 2–5) and 33 age- and sex-matched healthy controls. Laboratory parameters, including hemoglobin, ferritin, transferrin saturation (TSAT), EPO, ERFE, hepcidin, and renal indices (eGFR, BUN, creatinine), were analyzed. Group differences were assessed using ANOVA or Kruskal–Wallis tests with post hoc analyses, and trends were evaluated using the Jonckheere–Terpstra test. Age- and sex-adjusted correlations and multivariable linear regression identified independent associations with hemoglobin. **Results**: Patients with CKD were older (61.2 ± 14.8 vs. 33.4 ± 10.7 years, *p* < 0.001) and had lower hemoglobin (11.8 ± 1.9 vs. 13.5 ± 1.4 g/dL, *p* < 0.001) and higher ferritin levels (245 (110–470) vs. 105 (40–240) ng/mL, *p* = 0.002) compared with controls. eGFR declined progressively across CKD stages (median (IQR): 73 (64–86) to 12 (7–17) mL/min/1.73 m^2^, *p*-trend < 0.001). ERFE and hepcidin showed increasing trends with advancing CKD (*p*-trend = 0.031 and 0.047, respectively). Hemoglobin correlated negatively with ERFE (r = −0.40, 95% CI: −0.53 to −0.26, *p* < 0.001) and positively with eGFR (r = 0.42, 95% CI: 0.28–0.54, *p* < 0.001). In adjusted regression analysis, ERFE (β = −0.29, 95% CI: −0.41 to −0.18, *p* < 0.001) and eGFR (β = 0.25, 95% CI: 0.13–0.37, *p* < 0.001) remained independently associated variables of hemoglobin (adjusted R^2^ = 0.47). **Conclusions**: Anemia severity in CKD is independently associated with both renal dysfunction and higher ERFE concentrations, suggesting a disrupted ERFE–hepcidin regulatory balance. These findings provide hypothesis-generating insights into the complex interplay between iron metabolism and erythropoiesis in CKD. Validation in larger, multi-center longitudinal studies that include inflammatory markers is warranted.

## 1. Introduction

Chronic kidney disease (CKD) is a major global health problem affecting approximately 10% of the adult population worldwide [1,2]. Progressive loss of renal function not only leads to the accumulation of uremic toxins but also contributes to cardiovascular complications, mineral–bone disorders, and hematological abnormalities [3,4]. Among these complications, anemia is one of the most common and clinically significant.

Anemia in CKD has a multifactorial etiology. Reduced endogenous erythropoietin (EPO) production, disturbances in iron metabolism, chronic inflammation, and bone marrow suppression are the principal contributors [5,6,7,8]. Hepcidin, a hepatic peptide hormone, plays a central role in the regulation of systemic iron balance. By inhibiting iron absorption from enterocytes and iron release from macrophages, hepcidin ensures iron homeostasis. In CKD, increased inflammatory activity and reduced renal clearance result in elevated hepcidin levels, which in turn cause “functional iron deficiency” and aggravate anemia [9,10].

In recent years, erythroferrone (ERFE) has been identified as a novel hormone linking erythropoiesis and iron homeostasis. Secreted by erythroblasts, ERFE suppresses hepcidin synthesis and thereby facilitates iron mobilization during periods of increased erythropoietic activity. In healthy individuals, ERFE levels rise rapidly in response to erythropoietic stimulation, whereas hepcidin levels decrease [11,12]. However, in CKD, inflammation, insufficient erythropoietin production, and iron dysregulation may disrupt this ERFE–hepcidin axis. These alterations may be critical in determining the severity of anemia and the response to therapy.

Only a limited number of studies have simultaneously assessed ERFE, hepcidin, and EPO levels in CKD patients, and the available findings remain inconsistent [13,14]. Some reports suggest elevated ERFE levels, while others demonstrate no significant differences or weak correlations with hepcidin. Consequently, the role of the ERFE–hepcidin–EPO axis in the pathophysiology of CKD-related anemia has not been fully elucidated [15,16].

Recent evidence further highlights the potential of ERFE as a modulator of iron metabolism and erythropoiesis in CKD, yet its behavior appears highly context-dependent and influenced by inflammatory status and residual renal function [17,18,19,20]. Despite this, few studies have adjusted for these critical confounders, and none have simultaneously analyzed ERFE, hepcidin, and EPO using age- and sex-adjusted models. Furthermore, discrepancies in assay standardization and lack of data on inflammation-related biomarkers (e.g., CRP, IL-6) have limited the interpretability of previous findings [15,21,22].

The present study aimed to examine the associations rather than causal relationships among hemoglobin, hepcidin, ERFE, and EPO levels in patients with different stages of CKD and healthy controls, adjusting for age and sex, and to determine whether ERFE independently associates with hemoglobin levels after accounting for renal function and iron indices.

We hypothesized that higher ERFE concentrations would be independently associated with lower hemoglobin levels and reduced eGFR, reflecting a maladaptive activation of the ERFE–hepcidin axis in progressive CKD.

## 2. Materials and Methods

### 2.1. Study Design and Population

This study was designed as a cross-sectional, observational, case–control investigation conducted at the Nephrology Department of Düzce University Faculty of Medicine. A total of 159 participants were enrolled between January 2022 and December 2024, including 126 patients diagnosed with chronic kidney disease (CKD) and 33 healthy controls. The groups were not age-matched; therefore, all analyses were subsequently adjusted for age and sex.

CKD was staged according to the Kidney Disease: Improving Global Outcomes (KDIGO) guidelines, and patients were classified into stage 2 (n = 30), stage 3 (n = 35), stage 4 (n = 31), and stage 5 (n = 30). The control group consisted of individuals with normal renal function and no history of systemic disease.

**Figure 1:**
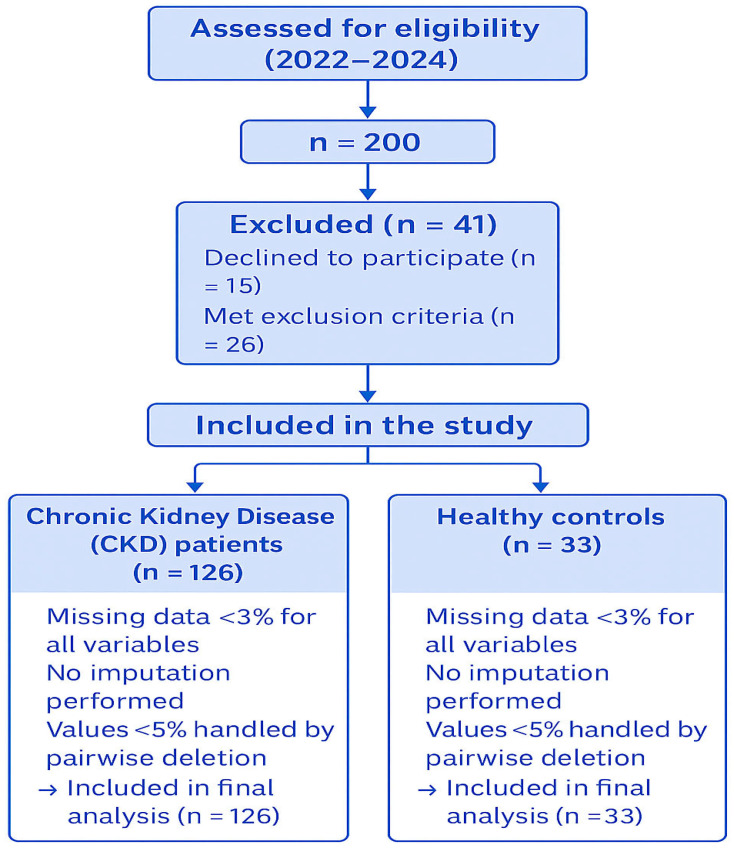
STROBE flow diagram of participant selection.

The flow of participants throughout the study is presented in Figure 1 according to STROBE guidelines.

### 2.2. Inclusion and Exclusion Criteria

Eligible participants were adults aged ≥18 years with a confirmed diagnosis of CKD for at least six months who provided written informed consent. Exclusion criteria were as follows: presence of active infection, history of malignancy, hematological or chronic inflammatory disorders, recent blood transfusion within the last three months, ongoing immunosuppressive therapy, or recent use of iron supplementation or erythropoiesis-stimulating agents. Pregnant or breastfeeding women were also excluded from the study.

Data on inflammatory biomarkers (C-reactive protein, interleukin-6), nutritional indicators (albumin, BMI), and medication history (ACE inhibitors, ARBs, SGLT2 inhibitors) were not consistently available and were therefore excluded from analysis; this limitation was addressed in Section 4.

### 2.3. Clinical and Laboratory Assessments

Detailed demographic and clinical data were collected from all participants, including age, sex, disease duration, and comorbidities. Peripheral venous blood samples were obtained after overnight fasting. Hemoglobin (Hb) levels, serum ferritin, transferrin saturation (TSAT), endogenous erythropoietin (EPO), hepcidin, and erythroferrone (ERFE) concentrations were measured using standardized laboratory techniques.

Renal function was assessed by serum creatinine, blood urea nitrogen (BUN), and estimated glomerular filtration rate (eGFR), which was calculated using the CKD-EPI equation. Complete blood count parameters, including white blood cell count (WBC), neutrophil and lymphocyte counts, neutrophil-to-lymphocyte ratio (NLR), red cell distribution width (RDW), hematocrit (Hct), and platelet counts, were also recorded.

Hepcidin and ERFE concentrations were quantified using commercially available enzyme-linked immunosorbent assay (ELISA) kits (Cloud-Clone Corp., Wuhan, China) according to the manufacturer’s protocols. Serum EPO was measured using a chemiluminescent immunoassay (Immulite 2000, Siemens Healthcare Diagnostics, Erlangen, Germany).

All ELISA assays were performed in a single batch using manufacturer-provided calibrators and internal quality controls to minimize inter-assay variability. Batch numbers and calibration curves were recorded.

All assays were performed in duplicate under standardized laboratory conditions to ensure reproducibility. Inter-assay and intra-assay coefficients of variation were maintained below 10%. Although these assays are validated for research use, differences between ELISA platforms may affect comparability with reference-standard methods, and this was acknowledged as a study limitation.

### 2.4. Ethical Considerations

The study protocol was reviewed and approved by the Non-Interventional Clinical Research Ethics Committee of Düzce University (approval date: 5 April 2021; approval number: 2021/108). All procedures were performed in accordance with the ethical principles of the Declaration of Helsinki. Written informed consent was obtained from all participants prior to study enrollment. The confidentiality of participant data was maintained throughout the study, and all laboratory analyses were carried out anonymously using coded identifiers.

### 2.5. Statistical Analysis

All statistical analyses were performed using IBM SPSS Statistics for Windows, Version 25.0 (IBM Corp., Armonk, NY, USA). Continuous variables were assessed for normality using the Shapiro–Wilk test and visual inspection of histograms. Normally distributed data were presented as mean ± standard deviation (SD), whereas non-normally distributed data were summarized as median and interquartile range (IQR).

Comparisons between two groups (CKD vs. controls) were conducted using the independent-sample *t*-test for normally distributed variables and the Mann–Whitney U test for non-normally distributed variables. For comparisons across multiple groups (CKD stages 2–5 and controls), one-way ANOVA or the Kruskal–Wallis test was applied as appropriate, followed by Tukey’s or Dunn’s post hoc test when global significance was observed.

Categorical variables were expressed as frequencies and percentages and compared using the chi-square or Fisher’s exact test when applicable. Monotonic trends across CKD stages were examined using the Jonckheere–Terpstra trend test. Correlations among hematologic and biochemical parameters (hemoglobin, TSAT, ferritin, EPO, hepcidin, ERFE, eGFR, BUN, and creatinine) were evaluated using Spearman’s correlation coefficients. Partial correlation analyses adjusted for age and sex were further performed to control for potential confounding.

To determine independently associated variables of hemoglobin levels, multivariable linear regression analyses were performed including ERFE, hepcidin, ferritin, eGFR, and EPO as biologically relevant covariates, with adjustment for age and sex. Variables with >5% missing data were excluded from the regression models, whereas missing data <5% were handled by pairwise deletion. The overall rate of missing data was below 3% for all parameters, and no imputation was performed.

Model assumptions (linearity, homoscedasticity, normality of residuals, and absence of multicollinearity) were verified by residual plots, the Kolmogorov–Smirnov test, the Breusch–Pagan test, and variance inflation factors (VIF < 2.5). Model performance was assessed using the coefficient of determination (R^2^), adjusted R^2^, F-statistic, and root mean square error (RMSE).

## 3. Results

According to Table 1, patients with chronic kidney disease (CKD) were significantly older than healthy controls (mean age 61.2 ± 14.8 vs. 33.4 ± 10.7 years, *p* < 0.001, 95% CI for difference: 23.8–32.1). The sex distribution was similar between groups (48.4% vs. 63.6% females, *p* = 0.18). The median disease duration among CKD patients was 6 years (IQR: 3–10). Serum vitamin B12 concentrations were slightly higher in CKD patients compared with controls (*p* = 0.041), whereas endogenous erythropoietin (EPO) levels showed no significant difference between groups (*p* = 0.68).

As shown in Table 2, renal function deteriorated progressively across CKD stages. Estimated glomerular filtration rate (eGFR) decreased significantly from controls to stage 5 (median [IQR]: 115 (92–136) to 12 (7–17) mL/min/1.73 m^2^, *p*-trend < 0.001). Parallel increases were observed in blood urea nitrogen (BUN) and serum creatinine (both *p*-trend < 0.001).

Hemoglobin concentrations declined gradually from early to advanced CKD (median [IQR]: 13.4 (12.5–14.8) in controls to 10.7 (9.5–11.9) g/dL in stage 5; *p*-trend < 0.001). Ferritin levels increased with disease severity (*p* = 0.002), while transferrin saturation (TSAT) showed no significant variation among stages (*p* = 0.59).

Hepcidin and erythroferrone (ERFE) levels demonstrated stepwise increases with CKD progression. Although intergroup comparisons did not reach conventional significance (*p* = 0.09 and *p* = 0.11, respectively), nonparametric trend analysis confirmed significant monotonic elevations across stages (hepcidin *p*-trend = 0.047; ERFE *p*-trend = 0.031).

According to Table 3, hemoglobin levels exhibited significant negative correlations with ERFE (r = −0.402, *p* < 0.001), BUN (r = −0.438, *p* < 0.001), and creatinine (r = −0.336, *p* < 0.001), and a positive correlation with eGFR (r = 0.421, *p* < 0.001). After adjusting for age and sex, these associations remained significant, indicating robust inverse relationships between hemoglobin and renal impairment markers.

ERFE was positively correlated with hepcidin (r = 0.517, *p* < 0.001) and inversely correlated with eGFR (r = −0.648, *p* < 0.001). Hepcidin correlated positively with BUN (r = 0.541, *p* < 0.001) and creatinine (r = 0.536, *p* < 0.001).

Multicollinearity was examined using variance inflation factors (VIFs), all of which were <2.0, indicating the absence of significant collinearity.

In multivariable regression modeling (Table 4), hemoglobin was entered as the dependent variable, and ERFE, hepcidin, ferritin, eGFR, and EPO were included as predictors with adjustment for age and sex.

ERFE (β = −0.29, 95% CI: −0.41 to −0.18, *p* < 0.001) and eGFR (β = 0.25, 95% CI: 0.13–0.37, *p* < 0.001) emerged as independently associated variables of hemoglobin levels. Hepcidin and ferritin demonstrated marginal associations (*p* = 0.09 and *p* = 0.07, respectively), while EPO showed no significant effect (*p* = 0.39). The model explained 46.8% of the variance in hemoglobin (adjusted R^2^ = 0.468), and residuals satisfied normality and homoscedasticity assumptions (Kolmogorov–Smirnov *p* = 0.41, Breusch–Pagan *p* = 0.37).

Also, significant correlations between hemoglobin levels and ERFE, hepcidin, and eGFR were observed (see Supplementary Figure S1).

Post hoc power analysis using G*Power version 3.1.9.7. indicated that with n = 159, α = 0.05, and the observed effect size (f^2^ = 0.22), the achieved power was 0.91, confirming adequate sample size for the primary model. Multicollinearity diagnostics showed all VIF values < 2.0 and tolerance > 0.5. Residual plots demonstrated random distribution without heteroscedasticity.

Collectively, the results suggest that both ERFE and eGFR independently associate with hemoglobin levels in CKD, while hepcidin and ferritin show weaker, borderline associations. No significant sex or age effects were observed after adjustment.

## 4. Discussion

This study investigated the relationships between hemoglobin, erythroferrone, hepcidin, erythropoietin, ferritin, transferrin saturation, and renal function markers across different stages of chronic kidney disease in comparison with healthy controls. The results demonstrated a progressive decline in hemoglobin levels and estimated glomerular filtration rate with advancing stages, accompanied by significant increases in blood urea nitrogen and serum creatinine. These findings are consistent with the well-established concept that the deterioration of renal function is accompanied by worsening anemia and biochemical alterations reflecting impaired clearance and metabolic disturbances.

In terms of demographic characteristics, patients were older than the control group, reflecting the higher prevalence of chronic kidney disease in older populations. Because age independently affects hemoglobin and renal parameters, subsequent analyses were adjusted for age and sex to minimize confounding. The sex distribution did not differ significantly, supporting the representativeness of the sample. Interestingly, vitamin B12 levels were significantly lower in patients compared with controls. Although vitamin B12 deficiency is not widely reported as a common complication of chronic kidney disease, reduced intake, impaired absorption, and comorbid conditions may contribute to this observation, as suggested in previous nutritional and nephrology studies [23,24,25,26].

Hemoglobin levels showed a stepwise reduction with disease progression, with the most pronounced decrease in stages 4 and 5. This observation aligns with prior reports indicating that anemia becomes more prevalent and severe in advanced stages of chronic kidney disease. Nevertheless, classical iron parameters such as ferritin and transferrin saturation did not differ significantly across groups [5,27]. This may reflect the limited sensitivity of these markers in the context of chronic inflammation and functional iron deficiency, a phenomenon frequently described in the literature. Ferritin is well recognized as an acute-phase reactant, and its elevation may mask true iron deficiency in chronic disease settings [28,29].

Hepcidin levels exhibited an increasing trend across stages, yet the differences did not reach statistical significance. This pattern became significant when trend analysis (*p*-trend < 0.05) was performed, supporting a gradual rise with declining eGFR. Most studies have shown higher hepcidin levels in patients with impaired renal function due to both decreased clearance and enhanced inflammatory activity. However, in the present cohort, the lack of inflammatory marker measurements (e.g., CRP, IL-6) limits interpretation of this finding [13,30]. Similarly, erythroferrone levels demonstrated a progressive increase from controls to advanced stages, but without statistical significance in overall comparison. In healthy physiology, erythroferrone is expected to rise in response to enhanced erythropoietic activity and suppress hepcidin synthesis [31]. In our study, however, erythroferrone correlated positively with hepcidin, which may reflect a disrupted erythroferrone–hepcidin axis in chronic kidney disease. This observation, rather than implying direct dysregulation, may simply reflect shared upregulation under the influence of disease severity or systemic inflammation. Previous studies have described this paradoxical relationship and suggested that inflammation and inadequate erythropoietin signaling may impair the normal regulatory feedback between these hormones [32].

Erythropoietin levels did not differ significantly across stages or between patients and controls. This finding does not contradict known pathophysiology; rather, it supports the concept of “relative EPO deficiency,” in which EPO production fails to rise proportionally to declining hemoglobin concentrations despite preserved renal secretion capacity [33]. These results highlight the heterogeneity of erythropoietin dynamics in chronic kidney disease and emphasize that erythropoietin levels alone may not adequately explain anemia severity [34,35].

Correlation analyses provided further insights. Hemoglobin levels were negatively correlated with erythroferrone and hepcidin, but positively with estimated glomerular filtration rate. These findings support the concept that worsening renal function contributes to anemia while suggesting that dysregulation of novel biomarkers may play a role in its pathogenesis. Importantly, partial correlation analysis confirmed that ERFE remained negatively associated with hemoglobin even after adjustment for age and sex, supporting its potential as an independent biomarker of anemia severity. The strong positive correlation observed between erythroferrone and hepcidin suggests that the classical inhibitory relationship may not operate normally in this setting. Several authors have proposed that this phenomenon represents an inappropriate erythroferrone response in the context of chronic kidney disease, in which the hormone fails to adequately suppress hepcidin despite rising levels [36].

In multivariate regression analysis, erythroferrone and estimated glomerular filtration rate emerged as independent determinants of hemoglobin levels. However, given the moderate multicollinearity between ERFE and eGFR (VIF < 2.0), these results should be interpreted with caution. This finding underlines the importance of both renal function and the erythroferrone–hepcidin axis in the development of anemia in chronic kidney disease. However, the modest explanatory power of the model indicates that additional factors, such as systemic inflammation, nutritional deficiencies, or bone marrow dysfunction, are also likely contributors. The absence of inflammatory markers such as C-reactive protein or interleukin-6 in the present dataset represents a limitation, as these could have provided a clearer picture of the interplay between inflammation, hepcidin, and erythroferrone [32,37,38].

The strengths of this study include the use of age- and sex-adjusted analyses, the inclusion of multiple CKD stages, and the simultaneous evaluation of traditional anemia markers and novel regulatory hormones, allowing for a comprehensive perspective on the progression of anemia in chronic kidney disease. On the other hand, limitations must be acknowledged. The study was conducted at a single center with a relatively limited sample size, which may restrict the generalizability of the findings. The cross-sectional design precludes causal inference, and the lack of CRP/IL-6 and nutritional data (albumin, BMI) may have confounded associations between ERFE and hepcidin. Additionally, differences in ELISA assay calibration and the use of research-grade kits (Cloud-Clone) could have introduced measurement variability.

## 5. Conclusions

In conclusion, this study indicates that anemia in chronic kidney disease is associated rather than causally driven by complex and possibly dysregulated interactions among erythroferrone, hepcidin, and erythropoietin. The consistent association of hemoglobin with renal function parameters reinforces the central role of kidney impairment, while the unexpected positive relationship between erythroferrone and hepcidin may reflect shared activation pathways rather than true physiological disruption. From a clinical perspective, these findings provide hypothesis-generating evidence that integrating ERFE and hepcidin measurements with traditional anemia indices could enhance understanding of iron metabolism and erythropoietic adaptation in CKD. Future multicenter, longitudinal studies incorporating inflammatory and nutritional markers are needed to validate these associations, explore mechanistic pathways, and clarify whether ERFE and hepcidin could serve as diagnostic or therapeutic targets in the management of CKD-related anemia.

## Figures and Tables

**Table 1:** Demographic and clinical characteristics of participants.

Variable	CKD (n = 126)	Control (n = 33)	*p*-Value	95% CI (Mean Difference)
Age (years, mean ± SD)	61.2 ± 14.8	33.4 ± 10.7	<0.001	23.8–32.1
Female, n (%)	61 (48.4)	21 (63.6)	0.18	—
CKD duration (years, median [IQR])	6 (3–10)	—	—	—
Vitamin B12 (pg/mL, median [IQR])	415 (210–720)	380 (230–640)	0.041	—
EPO (mIU/mL, median [IQR])	13.1 (4.1–47.6]	12.6 (4.3–31.3]	0.68	—

Values are expressed as mean ± SD or median [IQR]. CKD = chronic kidney disease; EPO = erythropoietin. *p* < 0.05 was considered statistically significant.

**Table 2:** Laboratory parameters across CKD stages and controls.

Parameter	Control (n = 33)	Stage 2 (n = 30)	Stage 3 (n = 35)	Stage 4 (n = 31)	Stage 5 (n = 30)	*p*-Value	*p*-Trend
eGFR (mL/min/1.73 m^2^)	115 (92–136)	73 (64–86)	48 (36–56)	26 (18–33)	12 (7–17)	<0.001	<0.001
BUN (mg/dL)	11 (7–18)	19 (11–25)	27 (19–45)	41 (25–80)	66 (39–112)	<0.001	<0.001
Creatinine (mg/dL)	0.8 (0.6–1.1)	1.1 (0.8–1.5)	1.7 (1.2–2.4)	2.9 (1.9–4.5)	5.2 (3.0–9.4)	<0.001	<0.001
Hemoglobin (g/dL)	13.4 (12.5–14.8)	13.0 (11.9–14.3)	12.2 (10.8–13.7)	11.3 (9.8–12.6)	10.7 (9.5–11.9)	<0.001	<0.001
TSAT (%)	22 (15–42)	25 (14–48)	27 (17–52)	24 (14–47)	23 (13–42)	0.59	0.33
Ferritin (ng/mL)	105 (40–240)	152 (70–320)	215 (90–460)	268 (110–590)	318 (150–750)	0.002	0.006
Hepcidin (ng/mL)	18 (12–24)	30 (20–41)	37 (25–60)	43 (28–75)	47 (30–88)	0.09	0.047
ERFE (ng/mL)	2.8 (1.1–6.3)	6.2 (3.5–9.8)	13.7 (6.9–25.4)	20.8 (10.3–31.7)	32.6 (20.1–46.3)	0.11	0.031

Values are presented as median [IQR]. CKD = chronic kidney disease; ERFE = erythroferrone; eGFR = estimated glomerular filtration rate; BUN = blood urea nitrogen; TSAT = transferrin saturation. *p* < 0.05 was considered statistically significant.

**Table 3:** Partial correlation analysis among hematologic and biochemical parameters.

Parameters	r (Partial)	95% CI	*p*-Value
Hemoglobin vs. ERFE	−0.402	−0.53 to −0.26	<0.001
Hemoglobin vs. Hepcidin	−0.115	−0.29 to 0.08	0.19
Hemoglobin vs. eGFR	+0.421	0.28 to 0.54	<0.001
ERFE vs. Hepcidin	+0.517	0.39 to 0.62	<0.001
ERFE vs. eGFR	−0.648	−0.73 to −0.53	<0.001
Hepcidin vs. BUN	+0.541	0.39 to 0.65	<0.001
Hepcidin vs. Creatinine	+0.536	0.38 to 0.64	<0.001

Values adjusted for age and sex. r = partial correlation coefficient. ERFE = erythroferrone; eGFR = estimated glomerular filtration rate; BUN = blood urea nitrogen.

**Table 4:** Multivariable linear regression analysis for determinants of hemoglobin levels.

**Independent Variable**	**β Coefficient**	**95% CI**	**SE**	***p*-Value**	**VIF**
ERFE (ng/mL)	−0.29	−0.41 to −0.18	0.07	<0.001	1.42
Hepcidin (ng/mL)	−0.10	−0.21 to 0.02	0.06	0.09	1.57
Ferritin (ng/mL)	−0.08	−0.17 to 0.01	0.05	0.07	1.33
eGFR (mL/min/1.73 m^2^)	+0.25	0.13 to 0.37	0.06	<0.001	1.68
EPO (mIU/mL)	−0.05	−0.16 to 0.06	0.06	0.39	1.22
Age (years)	−0.07	−0.18 to 0.03	0.05	0.15	1.19
Sex (female)	+0.03	−0.07 to 0.12	0.04	0.52	1.05

Dependent variable: hemoglobin (g/dL). Adjusted R^2^ = 0.468; F(7,151) = 20.54, *p* < 0.001. ERFE = erythroferrone; eGFR = estimated glomerular filtration rate; EPO = erythropoietin. *p* < 0.05 was considered statistically significant.

## Data Availability

The data presented in this study are available on request from the corresponding author. The data are not publicly available due to ethical and privacy restrictions.

## Supplementary Materials

The following supporting information can be downloaded at: https://www.mdpi.com/article/10.3390/jcm14217789/s1, Figure S1: Scatterplots showing correlations between hemoglobin and (A) ERFE, (B) hepcidin, and (C) eGFR. Regression lines and 95% confidence intervals are shown.

## Abbreviations

CKD	Chronic Kidney Disease
ERFE	Erythroferrone
EPO	Erythropoietin
TSAT	Transferrin Saturation
eGFR	Estimated Glomerular Filtration Rate
BUN	Blood Urea Nitrogen
Hb	Hemoglobin
Hct	Hematocrit
RDW	Red Cell Distribution Width
NLR	Neutrophil-to-Lymphocyte Ratio
WBC	White Blood Cell
