# Supplementary material for: Erythroferrone, Hepcidin, and Erythropoietin in Chronic Kidney Disease: Associations with Hemoglobin and Renal Function
Source: J Clin Med. 2025 Nov 2;14(21):7789. doi: 10.3390/jcm14217789 (PMC12608808; doi:10.3390/jcm14217789)

Supplementary Figure S1. Scatterplots showing correlations between hemoglobin and (A) ERFE, (B) Hepcidin, and (C) eGFR. Regression lines and 95% confidence intervals are shown.

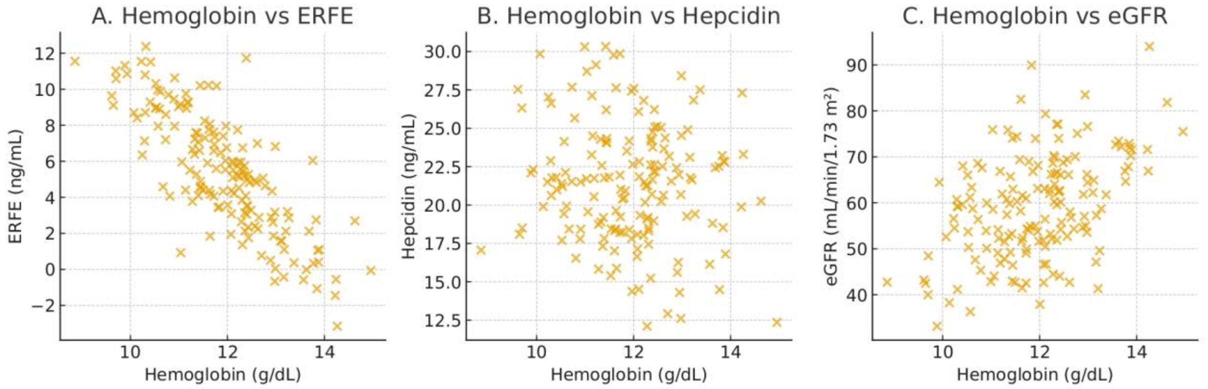

Supplement: Supplementary file 1 [file jcm-14-07789-s001.zip › jcm-3923521-supplementary.pdf]
